# Nasal shedding of vaccine viruses after immunization with a Russian‐backbone live attenuated influenza vaccine in India

**DOI:** 10.1111/irv.13149

**Published:** 2023-06-26

**Authors:** Lalit Dar, Anand Krishnan, Ramesh Kumar, Shivram Dhakad, Avinash Choudekar, Sumedha Bagga, Amrit Sharma, Amit Kumar, Jyoti Jethani, Siddhartha Saha, Ritvik Amarchand, Rakesh Kumar, Aashish Choudhary, Venkatesh Vinayak Narayan, Giridara Gopal, Kathryn E. Lafond, Stephen Lindstrom

**Affiliations:** ^1^ Department of Microbiology All India Institute of Medical Sciences New Delhi India; ^2^ Centre for Community Medicine All India Institute of Medical Sciences New Delhi India; ^3^ Influenza Division US Centers for Disease Control and Prevention New Delhi India; ^4^ Influenza Division US Centers for Disease Control and Prevention Atlanta Georgia USA

**Keywords:** developing countries, influenza, live attenuated influenza vaccine, pediatric, vaccine virus shedding

## Abstract

**Background:**

We present post‐vaccination nasal shedding findings from the phase IV, community‐based, triple‐blinded RCT conducted to assess efficacy of trivalent LAIV and inactivated influenza vaccines in rural north India.

**Methods:**

Children aged 2–10 years received LAIV or intranasal placebo across 2015 and 2016, as per initial allocation. On days 2 and 4 post‐vaccination, trained study nurses collected nasal swabs from randomly selected subset of trial participants based on operational feasibility, accounting for 10.0% and 11.4% of enrolled participants in 2015 and 2016, respectively. Swabs were collected in viral transport medium and transported under cold chain to laboratory for testing by reverse transcriptase real‐time polymerase chain reaction.

**Results:**

In year 1, on day 2 post‐vaccination, 71.2% (74/104) of LAIV recipients shed at least one of vaccine virus strains compared to 42.3% (44/104) on day 4. During year 1, on day 2 post‐vaccination, LAIV‐A(H1N1)pdm09 was detected in nasal swabs of 12% LAIV recipients, LAIV‐A(H3N2) in 41%, and LAIV‐B in 59%. In year 2, virus shedding was substantially lower; 29.6% (32/108) of LAIV recipients shed one of the vaccine virus strains on day 2 compared to 21.3% on day 4 (23/108).

**Conclusion:**

At day 2 post‐vaccination in year 1, two‐thirds of LAIV recipients were shedding vaccine viruses. Shedding of vaccine viruses varied between strains and was lower in year 2. More research is needed to determine the reason for lower virus shedding and vaccine efficacy for LAIV‐A(H1N1)pdm09.

## 1 INTRODUCTION

Seasonal influenza virus infections are an important contributor to severe acute lower respiratory infections (ALRI) in young children. Children in low income and lower middle‐income countries bear a disproportionate burden of influenza‐associated ALRI.^1^, ^2^ One of the effective ways to prevent influenza is vaccination. Presently, there exist three main types of licensed seasonal influenza vaccines, including the inactivated influenza vaccine (IIV), live attenuated influenza vaccine (LAIV), and the protein‐based recombinant influenza vaccine (RIV), each having their own benefits and limitations.^3^, ^4^

LAIVs are intranasally administered and contain a temperature‐sensitive attenuated virus that can efficiently replicate in the upper respiratory tract but not at the higher temperatures of the lower respiratory tract. LAIV viruses replicate in the nasopharynx, mimic a natural infection, and elicit a broad and robust immune response including cellular, humoral, and mucosal responses.^3^, ^4^, ^5^, ^6^, ^7^

LAIVs consist of reassortant viruses, which contain six internal gene segments (PB2, PB1, PA, NP, M, and NS) derived from the master donor viruses and hemagglutinin (HA) and neuraminidase (NA) gene segments from the seasonal viral strains. The internal genes contributed by the master donor viruses confer the cold‐adapted, temperature‐sensitive, and attenuated phenotypes. Two different LAIV technologies, Ann Arbor‐backbone and Russian‐backbone, are currently in use. While the Ann Arbor‐backbone LAIV, approved for use in the United States, is generated from the master donor viruses, A/Ann Arbor/6/60 and B/Ann Arbor/1/66, the Russian‐backbone LAIV, produced by the Serum Institute of India Pvt. Ltd., is based on A/Leningrad/134/17/57 (H2N2) and B/USSR/60/69.^4^, ^8^, ^9^, ^10^, ^11^ Given the advantages associated with LAIV, including the non‐invasive route of administration and high‐yielding manufacturing process, it is thought to be an attractive option for young children in resource‐poor settings, particularly during an influenza pandemic.^5^, ^11^, ^12^

Two randomized, double‐blind, placebo‐controlled trials conducted among children in Bangladesh and Senegal using the same vaccine based on the Russian backbone reported different results. While in Bangladesh the vaccine efficacy of LAIV against all influenza virus strains was 41.0% (95% CI 28.0 to 51.6), no efficacy was observed in the Senegal trial (0.0% [−26.4 to 20.9]), despite the use of the same lot of lyophilized vaccine in both studies.^13^, ^14^ The poor efficacy observed among children in Senegal was primarily attributed to the lack of protection against influenza A(H1N1)pdm09, the predominant circulating strain. The vaccine‐mismatched influenza B lineage could also have contributed to lower efficacy in those trials.^12^, ^14^ The lack of effectiveness of the Ann Arbor‐backbone LAIV against influenza A(H1N1)pdm09‐like viruses has also been seen in the US during the 2013–2014 and 2015–2016 seasons.^5^, ^12^, ^15^

The factors influencing the ability of LAIV viruses to bind, enter, and replicate within nasopharyngeal host cells may affect the subsequent immunogenicity and vaccine effectiveness. Post‐vaccination nasal shedding of vaccine viruses may be a surrogate indicator for appropriate maintenance of cold chain, viability of vaccine viruses, and the ability of individual vaccine viruses to replicate within the nasopharynx. Therefore, post‐vaccination nasal shedding can serve as a potential indicator of vaccine efficacy and may help elucidate mechanisms underlying efficacy of specific LAIV strains.^3^, ^7^, ^16^, ^17^

Shedding was not assessed in the aforementioned trial conducted in Bangladesh. However, in the Senegal study, at least one of the vaccine virus strains was shed nasally in 83% of LAIV recipients 2–4 days post‐vaccination with lower detection of LAIV‐A(H1N1)pdm09 (22%) as compared to LAIV‐A(H3N2) (52%) and LAIV‐B (66%).^13^, ^14^ In this context, we assessed nasal shedding of vaccine viruses among children aged 2–10 years in rural north India alongside a 2‐year, phase IV, community‐based, triple‐blinded, randomized controlled trial to assess the efficacy of the Russian‐backbone trivalent LAIV.

## 2 MATERIALS AND METHODS

### 2.1 Study design and vaccine administration

We conducted this study among a subset of participants of the 2‐year, multi‐arm, triple‐blinded, phase IV, randomized, placebo‐controlled trial (*CTRI/2015/06/005902*) conducted to assess the efficacy of LAIV and IIV among children 2–10 years of age in the Ballabgarh block of Faridabad district in the northern state of Haryana, India. Participants were randomly allocated to LAIV, intranasal placebo, IIV, or intramuscular control group in a ratio of 2:1:2:1. Children aged 2–10 years received a single dose of trivalent LAIV or intranasal placebo across two consecutive years, 2015 and 2016, as per the initial allocation. The details of the study have been published.^18^ Based on operational considerations, around 10% of nasal vaccine (LAIV or nasal placebo) were randomly selected to study the uptake of LAIV among the study participants.

Study products were the Russian‐backbone LAIV (Nasovac‐S) and a similar looking placebo provided by Serum Institute of India Pvt. Ltd. (SIIPL). The vaccine contained the World Health Organization (WHO)‐recommended Southern Hemisphere vaccine strains for the corresponding years; A/California/7/2009 (H1N1)pdm09‐like, A/Switzerland/9715293/2013 (H3N2)‐like, and B/Phuket/3073/2013 (Yamagata lineage)‐like reassortants were included in year 1. In year 2, Nasovac‐S contained A/California/7/2009 (H1N1)pdm09‐like, A/HongKong/4801/2014 (H3N2)‐like, and B/Brisbane/60/2008 (Victoria‐lineage)‐like reassortants.^19^, ^20^ A single 0.5 mL dose of either vaccine or placebo was administered equally into both nostrils. Vaccine cold chain was monitored using temperature loggers and diligently maintained during each stage from manufacturing to utility.

### 2.2 Sample collection and processing

On days 2 and 4 post‐administration of LAIV or intranasal placebo, trained study nurses collected nasal swabs from a 10% random subset of enrolled participants in 2015 and 2016. From the list of children who were administered nasal vaccines (or nasal placebo), the epi info software (CDC, USA) was used to randomly select children for sampling. Nasal swabs were collected in viral transport medium and transported under cold chain to the Virology laboratory, Department of Microbiology, AIIMS, New Delhi. Unpaired and improperly collected samples were excluded from further analysis. The samples were processed in a class II biological safety cabinet following standard precautions. Following centrifugation at 4°C, the supernatants were aliquoted into sterile cryovials; one vial was used for nucleic acid isolation, and the remaining was stored at −80°C.

### 2.3 Nucleic acid extraction and real‐time reverse transcription polymerase chain reaction (rRT‐PCR)

Viral RNA was extracted from a 50 μL volume of each sample using the MagMax™‐96 Viral Isolation kit (Thermo Fisher Scientific, USA), according to the manufacturer's instructions. Subsequently, to detect wild‐type Influenza A and B viruses and vaccine viruses (LAIV‐A and B), one‐step real‐time reverse‐transcription PCR (rRT‐PCR) was performed using specific oligonucleotide primer sets and probes provided by the Influenza Division of the Centers of Disease Control and Prevention (CDC), Atlanta, Georgia through the International Resource Reagent (https://www.internationalreagentresource.org) (information available from CDC upon request: clsis@cdc.gov). Specifically, the presence of vaccine virus was identified by rRT‐PCR using oligonucleotide primers and probes designed to detect internal virus genes specific to the A/Leningrad/134/17/57 (H2N2) and B/USSR/60/69 master donor viruses that were utilized to create the cold‐adapted reassortants contained in the LAIV. Influenza A positive samples were further subtyped into A(H3N2) and A(H1N1)pdm09 viruses.

The 25 μL PCR reaction composed of 12.5 μL 2X Agpath‐ID™ One‐Step RT‐PCR buffer (Applied Biosystems™, USA), 0.5 μL of 10 μM forward and reverse primers (target gene‐specific), 0.5 μL of 5 μM target‐specific probe, 1.0 μL of 25X RT‐PCR enzyme mix from Agpath‐ID™ One Step RT‐PCR kit, 5 μL of sample RNA, and 5.0 μL nuclease‐free water. The amplification process was performed in a StepOnePlus™ Real‐Time PCR System or an ABI 7500 Real‐Time PCR System (Applied Biosystems™, USA) according to the CDC protocol: 50°C for 30 min (reverse transcription) and 95°C for 10 min (DNA polymerase activation), followed by 45 cycles of 95 °C for 15 s (denaturation) and 55°C for 60 s (annealing and extension). The cycle threshold (Ct) value of each run reaction was normalized to the mean Ct of the pooled influenza positive control (CDC, USA) across all runs. Results were interpreted as per the protocol of CDC, USA.

We expressed nasal shedding proportion among those who received the vaccine or placebo on days 2 and 4 for each year. We also looked at viral shedding by influenza type and subtypes.

## 3 RESULTS

The trial enrolled 1015 children in the LAIV group and 507 children in the intra‐nasal placebo group in the first year. The nasal shedding analysis was done on specimens from 104 (10.2%) children from the LAIV group and 48 (9.5%) children from the intranasal placebo group in the first year. In the second year, a total of 967 children were administered LAIV, and 477 children were administered intranasal placebo, as per the initial allocation. Nasal shedding study was done on specimens from 108 (11.2%) children in the LAIV group and the 56 (11.7%) in the intranasal placebo group.

Except for one child in the placebo group shedding LAIV‐A(H3N2) on day 4 in the first year, no nasal shedding of vaccine or wild‐type virus was seen in this group in the 2 years of the study. Neither circulating virus nor vaccine virus was detected in 23.1% of LAIV recipients on day 2 and in 52.9% on day 4 of year 1. These proportions were 61.1% on day 2 and 76.9% on day 4 of year 2.

Wild‐type influenza viruses were excreted in a comparable proportion on day 2 post‐immunization of both the study years (8.7% in year 1 and 9.3% in year 2). On day 4 post‐immunization, 7.7% were shedding wild viruses in the first year as compared to 1.9% in the second year. Shedding of both vaccine and wild‐type virus by the same person was seen in only three children in year 1. During year 1, 71.2% of LAIV recipients shed any LAIV virus on day 2, which was reduced to 42.3% on day 4. In year 2, LAIV detection was markedly reduced to 29.6% on day 2 and 21.3% on day 4 (Table 1).

**TABLE 1:** Nasal shedding of influenza viruses (LAIV and wild) among recipients of LAIV vaccine aged 2–10 years in rural north India, 2015–2016.

	Year 1 (*n* = 104)		Year 2 (*n* = 108)	
	2 days post‐immunization *n* (proportion [95% CI])	4 days post‐immunization *n* (proportion [95% CI])	2 days post‐immunization *n* (proportion [95% CI])	4 days post‐immunization *n* (proportion [95% CI])
LAIV virus(es) only	71 (68.3% [58.6–76.6])	41 (39.4% [30.4–49.2])	32 (29.6% [21.8–39.0])	23 (21.3% [14.6–30.1])
LAIV and wild‐type virus(es)	3 (2.9% [0.9–8.7])	3 (2.9% [0.9–8.7])	0	0
Wild‐type virus(es) alone	6 (5.8% [2.6–12.4])	5 (4.8% [2.0–11.1])	10 (9.3% [5.0–16.4])	2 (1.9% [0.5–7.2])
No virus detected	24 (23.1% [15.9–32.3])	55 (52.9% [43.2–62.4])	66 (61.1% [51.6–69.9])	83 (76.9% [67.9–83.9])

The shedding of vaccine virus subtypes varied between the 2 days of surveillance in year 1. Specifically, on day 2 post‐vaccination, LAIV‐A(H1N1)pdm09 was detected in 11.5% of the LAIV recipients; this was lower than the number of LAIV recipients found to shed LAIV‐A(H3N2) (41.4%) and LAIV‐B (58.7%). Furthermore, on day 4 post‐vaccination, LAIV‐A(H3N2) and LAIV‐B were detected in 23.1% and 26.0% of LAIV recipients, respectively; however, no LAIV recipients (0.0%) had evidence of LAIV‐A(H1N1)pdm09. Only 7.7% of the LAIV recipients shed all three vaccine viruses nasally, and this was only on day 2 of the first year (Tables 2 and 3).

**TABLE 2:** Nasal shedding of LAIV virus(es) by type/subtype among recipients of LAIV vaccine aged 2–10 years in rural north India, 2015–2016.

	Year 1 (*n* = 104)		Year 2 (*n* = 108)	
	2 days post‐immunization *n* (proportion [95% CI])	4 days post‐immunization *n* (proportion [95% CI])	2 days post‐immunization *n* (proportion [95% CI])	4 days post‐immunization *n* (proportion [95% CI])
LAIV virus type/subtype shed				
A(H1N1)pdm09	12 (11.5% [6.6–19.3])	0	4 (3.7% [1.4–9.5])	1 (1.0% [0.1–6.3])
A(H3N2)	43 (41.4% [32.2–51.1])	24 (23.1% [15.9–32.2])	8 (7.4% [3.7–14.2])	2 (1.9% [0.5–7.2])
B	61 (58.7% [48.9–67.8])	27 (26.0% [18.4–35.3])	26 (24.1% [16.9–33.1])	22 (20.4% [13.8–29.1])
Number of LAIV virus types/subtypes shed				
All 3 LAIV viruses	8 (7.7% [3.9–14.7])	0	0	0
≥2 LAIV viruses	34 (32.7% [24.3–42.4])	8 (7.7% [3.9–14.7])	6 (5.6% [2.5–11.9])	2 (1.9% [0.5–7.2])
≥1 LAIV virus	74 (71.2% [61.6–79.1])	44 (42.3% [33.1–52.1])	32 (29.6% [21.8–39.0])	23 (21.3% [14.6–30.1])

**TABLE 3:** Nasal shedding of influenza viruses among recipients of LAIV vaccine aged 2–10 years in rural north India, 2015–2016.

	Year 1 (*n* = 104)		Year 2 (*n* = 108)	
	2 days post‐immunization	4 days post‐immunization	2 days post‐immunization	4 days post‐immunization
LAIV virus(es) shed nasally				
LAIV‐A(H1N1)pdm09 + LAIV‐A(H3N2) + LAIV‐B	8 (7.7%)	0	0	0
LAIV‐A(H1N1)pdm09 + LAIV‐A(H3N2)	3 (2.9%)	0	4 (3.7%)	1 (1%)
LAIV‐A(H1N1)pdm09 + LAIV‐B	1 (1%)	0	0	0
LAIV‐A(H3N2) + LAIV‐B	22 (21.2%)	7 (6.7%)	2 (1.9%)	1 (1%)
LAIV‐A(H1N1)pdm09 only	0	0	0	0
LAIV‐A(H3N2) only	10 (9.6%)	15 (14.4%)	2 (1.9%)	0
LAIV‐A (untyped) + LAIV‐B	0	1 (1%)	0	0
LAIV‐B only	27 (26.0%)	18 (17.3%)	24 (22.2%)	21 (19.4%)
LAIV and wild‐type virus(es) shed nasally				
LAIV‐B + Wild‐type A(H3N2)	3 (2.9%)	1 (1%)	0	0
LAIV‐A(H3N2) + Wild‐type B	0	2 (1.9%)	0	0
Wild‐type virus(es) alone	6 (5.8%)	5 (4.8%)	10 (9.3%)	2 (1.9%)
No virus detected	24 (23.1%)	55 (52.9%)	66 (61.1%)	83 (76.9%)

As observed during year 1, nasal shedding of LAIV viruses was found to vary by strain in year 2, with A(H1N1)pdm09 detection being the least and influenza B being the most commonly detected. To elaborate, on day 2 post‐immunization, the detection of LAIV‐A(H1N1)pdm09 (3.7%) in LAIV recipients was lower than LAIV‐A(H3N2) (7.4%) and LAIV‐B (24.1%). Notably, only one (1%) LAIV recipient each was found to shed with LAIV‐A(H3N2) with LAIV‐A(H1N1)pdm09 and LAIV‐B, respectively, by day 4 post‐immunization; however, 19.4% of LAIV recipients were shedding LAIV‐B even on day 4 (Tables 2 and 3).

There were 15 children who had been sampled for shedding on days 2 and 4 for both years 1 and 2 (Table 4). Among these, three children did not shed any virus on any of the days during both years. Six children shed the virus during first year, while similar to the overall pattern of lower shedding in year 2, none of these six children shed viruses in year 2. However, there was one child who had shed in year 2 but not in year 1. Among these 15 children, 11 children shed LAIV B, six shed LAIV A(H3N2), and only two shed LAIV A(H1N1)pdm09 during any of the 2 years of study. Shedding of influenza A(H3N2) was also detected for two children during year 1.

**TABLE 4:** Nasal shedding of influenza viruses among 15 recipients of LAIV vaccine who got sampled during both years (2015 and 2016).

	YEAR 1			YEAR 2		
S. No.	LAIV‐A (H3N2)	LAIV‐A (H1N1)09pdm	LAIV‐B	LAIV‐A (H3N2)	LAIV‐A (H1N1)09pdm	LAIV‐B
Child 1	+	−	−	−	−	+
Child 2	−	−	+	−	−	+
Child 3	+	−	−	+	−	+
Child 4	−	−	+	−	−	+
Child 5	−	−	+	−	−	−
Child 6	−	−	+	−	−	−
Child 7	−	−	+	+	+	−
Child 8	−	−	−	−	−	+
Child 9	+	+	−	−	−	−
Child 10	+	−	+	−	−	−
Child 11	−	−	+	−	−	−
Child 12	+	−	+	−	−	−
Child 13	−	−	−	−	−	−
Child 14	−	−	−	−	−	−
Child 15	−	−	−	−	−	−

## 4 DISCUSSION

This study evaluated post‐vaccination nasal shedding of LAIV viruses among children aged 2–10 years during the 2‐year, phase IV, community‐based, triple‐blinded, randomized, placebo‐controlled trial primarily designed to assess vaccine efficacy, which was conducted in rural north India. Our key findings were high post‐vaccine nasal shedding in year 1, and reduced shedding in year 2, lower shedding for A(H1N1)pdm09 compared to the other viruses in both years, and higher shedding of influenza B virus.

We observed that in year 1, on day 2 post‐vaccination, about 70% of included LAIV recipients shed at least one vaccine virus strain. Since mucociliary clearance usually occurs within minutes, shedding at day 2 is likely due to viral replication in the nasopharynx, indicating vaccine virus viability.^21^ These results were concordant with the viral shedding findings reported in Senegal where, on day 2 post‐immunization, vaccine virus of at least one strain was detected in 74% of LAIV recipients.^14^ Also, in a phase II, randomized, placebo‐controlled trial conducted among children in Bangladesh evaluating a Russian‐backbone LAIV for shedding, on day 2 post‐immunization, vaccine virus of at least one strain was detected in 72% of LAIV recipients.^22^ Similar to our study, in both of these trials, detection of vaccine viruses was highest at day 2 post‐vaccination and decreased by day 4 in the Senegal (59%) and Bangladesh trials (64.7%).^14^, ^22^

The reduction of nasal shedding from 68.3% on day 2 of the first year to 29.6% in year 2 might be attributed to suppressed replication of LAIV viruses due to previous receipt of LAIV in year 1. Pre‐existing immunity is thought to influence LAIV viral recovery, and studies support the idea that viral shedding declines with repeated use of LAIV.^5^, ^23^, ^24^, ^25^

Low shedding of the A/California/7/2009 (H1N1)pdm09‐like virus seems to be a common observation among various studies. We observed that during year 1, 2 days post‐immunization, there was evidence of LAIV‐A(H1N1)pdm09 in nasal swabs of about 12% LAIV recipients. This was consistent with what was observed in the Senegal trial, where LAIV‐A(H1N1)pdm09 was detected in 19% of LAIV recipients.^14^ Notably, in the aforementioned phase II Bangladesh trial, no child shed LAIV‐A(H1N1)pdm09 vaccine strain.^22^

In our study, shedding varied among strains; during year 1, 2 days post‐vaccination, the proportion of children with LAIV‐B virus shedding was highest, followed by LAIV‐A(H3N2) and LAIV‐A(H1N1)pdm09. This was also observed in the Senegal trial (LAIV‐B [52%] > LAIV‐A(H3N2) [48%] > LAIV‐A(H1N1)pdm09 [19%]) and the phase II Bangladesh trial (LAIV‐B [59.3%] > LAIV‐A(H3N2) [40.0%] > LAIV‐A(H1N1)pdm09 [0.0%]).^14^, ^22^

While examining individual level data of 15 individuals who were sampled for shedding in both years (Table 4), overall shedding was higher on day 1 as compared to day 2 and higher in year 1 as compared to year 2. LAIV‐B was the most frequently shedded virus among these children. One of the patients in the placebo group had shed the LAIV A (H3N2) on day 4 post‐vaccination in year 1. This child was part of a household cohort in which there were four children who were enrolled in the study: One child received LAIV, one received IPV, and remaining two received nasal placebo. However, it is unclear as to why only one child having received a placebo still had shed the vaccine virus, as households having children allocated to different arms was common in our study area.

Viral shedding findings reported in this study were part of a randomized controlled trial evaluating the efficacy of a Russian‐backbone trivalent LAIV among children in rural North India. Influenza vaccine administered in year 2 had different strains of influenza A (H3N2) and influenza B viruses as compared to year 1. As per WHO southern hemisphere vaccine recommendation in Year 1, vaccines included influenza A/Switzerland/9715293/2013 (A(H3N2)‐like) and influenza B/Phuket/3073/2013 (Yamagata‐like) viruses. In year 2, LAIV included influenza A/Hong Kong/4801/2014 (A(H3N2)‐like) and influenza B/Brisbane/60/2008 (Victoria‐like) viruses. In both years, LAIV demonstrated significant vaccine efficacy against influenza A(H3N2) virus and matched influenza B viruses. However, LAIV had no statistically significant efficacy against influenza A(H1N1)pdm09 virus in either year.^18^ Although the nasal shedding results seem to corroborate efficacy results in our study, vaccine virus shedding in humans does not adequately predict the clinical efficacy of LAIV across trials.^22^

Several hypotheses have been proposed as potential reasons underlying reduced LAIV effectiveness against A(H1N1)pdm09‐like viruses. Importantly, reduced replicative fitness of the A/California/7/2009 (H1N1)pdm09‐like strains was suggested as the most likely reason underlying decreased vaccine effectiveness in the United States during the 2013–2014 and 2015–2016 influenza seasons. Reduced replicative fitness of the A(H1N1)pdm09 component may have led to low efficacy of LAIV observed in Senegal.^26^, ^27^, ^28^, ^29^, ^30^, ^31^ Subsequently, novel assays assessing replicative fitness were incorporated into the A(H1N1) strain selection process for the 2017–2018 influenza season, and a new H1N1 strain (A/Slovenia/2903/2015), an A/Michigan/45/2015 (H1N1)pdm09‐like virus, that demonstrated improved replication in primary human nasal epithelial cells, as compared to the A/Bolivia strain, and an ability to sustain multiple rounds of replication was included in LAIV4, substituting A/Bolivia/559/2013.^16^, ^26^, ^32^, ^33^, ^34^, ^35^

Since efficacy of LAIV has varied considerably across different settings, there exists a need for testing in different populations. To our knowledge, our study is the first comprehensive analysis of nasal shedding of vaccine viruses post‐immunization with a Russian‐backbone trivalent LAIV among children in India. Although information about nasal shedding of vaccine viruses is predictive of replicative fitness, it would be valuable to support the clinical results with in vitro findings. Specifically, replication of vaccine strains could be analyzed in primary human nasal epithelium air‐liquid cultures, a physiologically relevant model for respiratory research.^26^ Furthermore, pre‐pandemic A(H1N1) LAIV strains from seasons when the vaccine was effective against A(H1N1) influenza viruses better supported multiple rounds of replication than the 2013–2014 (A/California7/2009) and 2015–2016 (A/Bolivia/559/2013) formulations of LAIV4. Accordingly, it has been proposed that studies prior to inclusion of the A(H1N1)pdm09 component in LAIV4, infectivity of vaccine viruses should be evaluated by both the 50% tissue culture infective dose method, which measures the spread of vaccine virus between cells through sustained replication cycles, and the fluorescent focus assay, which measures infectivity of vaccine viruses through expression of antigen on cell surface.^16^, ^26^, ^27^ It would be valuable to utilize similar procedures to assess infectivity of the vaccine viruses incorporated in the Russian‐backbone trivalent LAIV utilized in this trial.

Although this is a post hoc analysis and not powered for this outcome, our analyses have attempted to further the understanding of mechanisms underlying varied efficacy of LAIV in developing countries and underscore the importance of assessing replicative fitness while selecting candidate vaccine viruses for inclusion in LAIV. More research is required to further determine the possible reasons that could explain lower virus shedding and vaccine efficacy for LAIV‐A(H1N1)pdm09 across different settings.

## AUTHOR CONTRIBUTIONS

**Lalit Dar**: Conceptualization (lead); investigation (lead); supervision (lead); validation (lead); writing—original draft (lead). **Anand Krishnan**: Conceptualization (equal); funding acquisition (lead); project administration (equal); resources (equal); writing—review and editing (equal). **Ramesh Kumar**: Data curation (equal); formal analysis (equal); investigation (equal); project administration (equal); supervision (equal); validation (equal). **Shivram Dhakad**: Investigation (equal); project administration (equal); validation (equal). **Avinash Choudekar**: Investigation (equal); project administration (equal). **Sumedha Bagga**: Investigation (equal); project administration (equal); validation (equal); writing—original draft (supporting). **Amrit Sharma**: investigation (supporting); project administration (supporting). **Amit Kumar**: Investigation (supporting); project administration (supporting). **Jyoti Jethani**: Investigation (supporting); project administration (supporting). **Siddhartha Saha**: Conceptualization (equal); funding acquisition (equal); resources (equal); writing—review and editing (equal). **Ritvik Amarchand**: Project administration (equal); resources (equal); writing—review and editing (supporting). **Rakesh Kumar**: Data curation (supporting); project administration (equal); writing—review and editing (equal). **Aashish Choudhary**: Investigation (supporting); project administration (supporting); supervision (supporting); writing—original draft (supporting). **Venkatesh Vinayak Narayan**: Data curation (equal); formal analysis (equal); project administration (supporting); software (equal); writing—review and editing (supporting). **Giridara Gopal**: Data curation (supporting); project administration (supporting). **Kathryn E. Lafond**: Conceptualization (equal); methodology (equal); writing—review and editing (equal). **Stephen Lindstrom**: Methodology (equal); resources (equal); writing—review and editing (equal).

## CONFLICT OF INTEREST STATEMENT

None declared.

### PEER REVIEW

The peer review history for this article is available at https://www.webofscience.com/api/gateway/wos/peer-review/10.1111/irv.13149.

## DISCLAIMER

The findings and conclusions of this report are those of the authors and do not necessarily represent the official position of All India Institute of Medical Sciences, New Delhi and US Centers for Disease Control and Prevention, Atlanta.

## Data Availability

The data that support the findings of this study are available from the corresponding author upon reasonable request.
